# Ayurvedic management of persistent ulcers after snakebite – A case report

**DOI:** 10.1016/j.jaim.2025.101228

**Published:** 2025-10-11

**Authors:** Sandesh Kumar, Sukanya Bhat

**Affiliations:** Department of PG Studies in Swasthavritta and Yoga Shri Dharmasthala Manjunatheshwara College of Ayurveda, Hospital and Research centre, Udupi, India

**Keywords:** Snake bite, Ayurveda, Wound, Ulcer, Inflammation

## Abstract

Snake bites pose a major public health challenge worldwide, with significant morbidity and mortality. Non-healing ulcers are common in snakebite survivors, often resulting in chronic pain, infection, necrosis, and, in severe cases, amputation. This case study discusses the Ayurvedic treatment of a non-healing ulcer following a viper bite in a 63-year-old woman. The treatment protocol included *Vrana Prakshalana* (ulcer washing), *Pariseka* (irrigation), and *Bandha* (bandaging) with medicated oils and pastes, along with oral traditional formulations like *Gandhaka Rasayana* and *Mahamanjishtadi kashaya*. Over four months, the patient experienced complete ulcer healing, pain reduction, and resolution of edema, illustrating the effectiveness of Ayurvedic therapies in addressing complex post-snakebite complications and preventing further issues. This case underscores Ayurveda's potential in managing chronic, treatment-resistant wounds.

## 1 Introduction

**Fig. 1:**
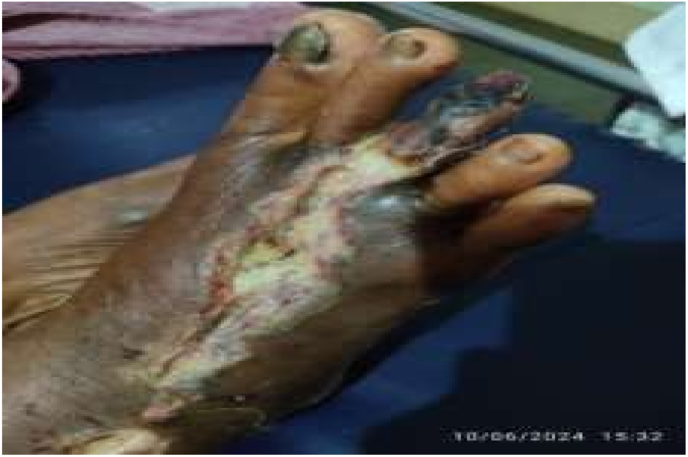
Ulcer at the time of admission.

**Fig. 2:**
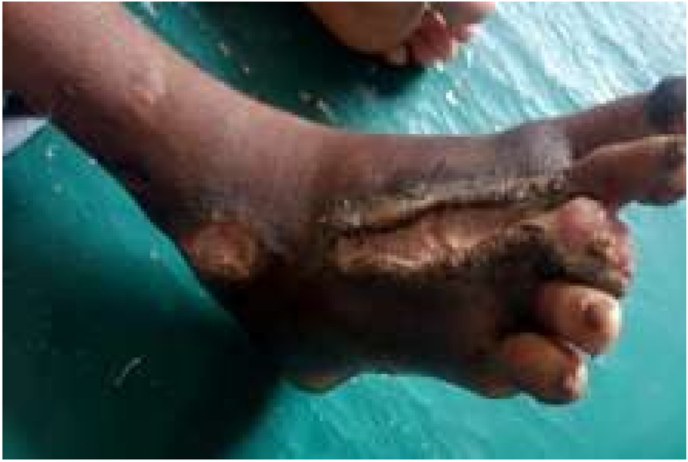
At the end of second month.

**Fig. 3:**
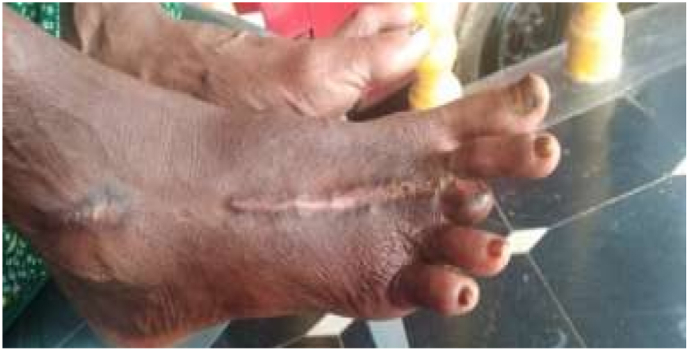
At the end of full course treatment.

**Fig. 4:**
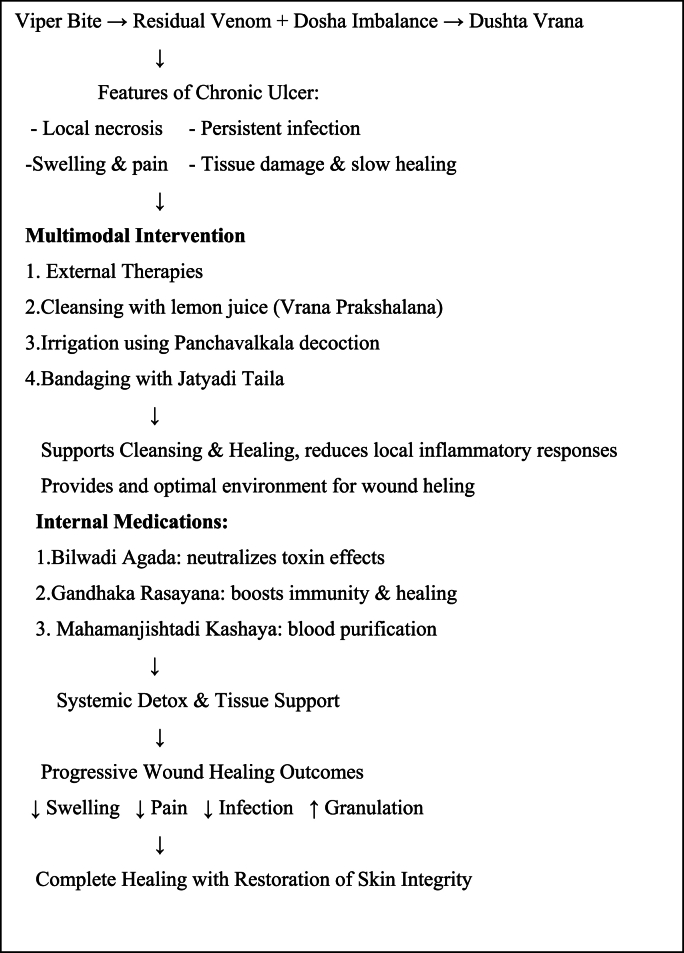
Flow chart of discussion.

Snake bites affect between 1.8 and 2.7 million individuals globally each year, and are responsible for an estimated 80,000 to 138,000 fatalities. Survivors of snake bites may also face long-term physical consequences, including amputations, paralysis, disability, and psychological issues [1]. The clinical presentation of snakebites can vary significantly, influenced by factors such as the species of snake, the quantity and potency of venom injected, the bite's location, and the patient's age and underlying health conditions [2]. Viper bites, particularly from Russell's viper (Daboia russelii), are a significant public health concern in India, accounting for a substantial proportion of snakebite incidents. These bites often result in severe local tissue damage, leading to complications such as necrosis, cellulitis, and chronic non-healing ulcers. A study from North Kerala reported that 19.5 % of patients bitten by Russell's viper developed local cellulitis, necrosis, or gangrene, with a mortality rate of 13.5 %. Challenges in managing viper bite ulcers include delayed presentation to healthcare facilities, inadequate wound care, and limited access to antivenom. These factors underscore the need for integrated treatment approaches that combine prompt medical intervention with supportive therapies to enhance wound healing and reduce morbidity [3] (see Fig. 1, Fig. 2, Fig. 3, Fig. 4)

Non-healing ulcers are a common sequel in individuals who survive snakebites. The severity of these ulcers is directly related to the potency of the venom involved. Common signs include swelling at the site of the bite and the spread of cellulitis. In cases of recovery, some patients may experience necrosis of the skin, muscles, tendons, and even bones. Secondary infections can also lead to purulent discharge and gangrene, which may necessitate amputation. To avoid further complications, it is crucial to manage these secondary infections caused by a variety of bacteria [4]. Traditional approaches to treating ulcers typically include infection control, promoting ulcer healing, surgical intervention, and amputation; however, these methods come with limitations and potential complications. This underscores the need for alternative treatments, such as those based on Ayurvedic principles.

Ayurveda has addressed the management of complications following viper (*Mandali sarpa*) bites, employing techniques such as *vrana prakshalana* (Washing the ulcer) *Pariseka*(irrigation) [5]. Chronic ulcers (*Dushta Vrana*) following snakebites are described in classical texts as being difficult to heal due to vitiated *Tridosha* (the three *doshas* in the body). These ulcers often present with persistent discharge (*Srava*), foul-smelling necrotic tissue (*Putipuya Mamsa*), pain (*Vedana*), and a long duration of existence (*Dirgha Kala Anubandhi*) [6]. Such ulcers are categorized as *Krichhra Sadhya*, meaning they are challenging to treat [7].

The classical Ayurvedic texts describe the *Sapta Upakrama* [8](seven therapeutic procedures) for managing *Dushta Vrana* to facilitate healing without further complications. These procedures are designed to debride necrotic tissue, maintain a favorable moist environment for ulcer healing, and optimize the nutritional, metabolic, and circulatory health of the affected tissues.

## 2 Patient information

A 63-year-old female housewife presented to the outpatient department with a complaint of a non-healing ulcer on the dorsum of her right foot, which had been persisting for 15 days. The ulcer was associated with swelling around the wound, a pricking-type pain, loss of sensation in the middle toe of the right foot, and blackish discoloration. According to her history, she had been bitten by a snake 15 days earlier while cutting grass in the field. Following the bite, she sought immediate medical attention at a nearby hospital, where she experienced pain, burning sensations, and bleeding at the bite site. The hospital confirmed that the bite was from a viper and administered treatment. However, she was uncertain whether anti-snake venom had been given during her treatment. After two days, she was discharged with oral analgesics and antibiotics. Despite performing daily wound dressings, she noticed no significant healing and no reduction in the size of the ulcer after 15 days. As a result, she decided to seek Ayurvedic treatment for further management and was admitted for further care.

### 2.1 Clinical findings

On general examination, no lymphadenopathy was noted, and all vital signs were found to be within normal limits. On local examination of the ulcer, it was observed that the margin was irregular and inflamed, extending from the third toe to the lateral malleolus. The tip of the third toe exhibited necrosis, and gangrenous changes were seen in the middle toe, which had a punched-out, sloping edge. The floor of the ulcer was covered with slough. Decreased pain perception was noted around the ulcer area. Hematological investigations were not repeated as they had already been conducted at the allopathic hospital.

## 3 Diagnostic assessments

Based on the patient's history, clinical signs, symptoms, and local examination findings, the case was diagnosed as *Visha Janya Dushta Vrana* (non-healing ulcer resulting from a venomous bite). The patient was advised to be admitted for further treatment. A treatment plan was developed, focusing on *Visha Hara Chikitsa* (anti-poison therapy), along with *Vrana Shodhana* and *Ropana* (wound cleansing and healing) to facilitate the healing process of the ulcer.

## 4 Therapeutic intervention

During treatment *Vrana Prakshalana* (washing the ulcer) with Lemon juice. Vrana Prakshalana is carried out by cleansing the ulcer with freshly prepared, undiluted lemon juice, which is poured directly over the wound using a sterile syringe or bowl. This is done once daily before dressing, as the lemon juice aids in microbial decontamination, reduces local pH, and facilitates slough removal. *Panchavalkala kashaya Pariseka*(irrigation) which involves the continuous and gentle irrigation of the wound using a freshly prepared and filtered lukewarm Panchavalkala decoction, maintained at approximately 37–40 °C. The decoction is poured steadily over the wound for 15–20 minutes using a vessel and the procedure is repeated once daily to reduce inflammation, and support healing. *Jatyadi taila* for *Bandha*(bandage) were used for clear the ulcer. Capsule Grab, *Bilwadi agada gulika*, *Gandhaka Rasayana*, *Mahamanjishtadi kashaya* were advised orally(Table .1). Though certain ingredients like *Gandhaka, Triphala, Guggulu* are repeated across multiple formulations their pharmacological action differ based on processing, formulation matrix and therapeutic context. Repeated use may enhance synergistic effects such as immunomodulation, detoxification or wound healing without leading to toxicity, provided therapeutic doses are maintained.

**Table 1:** Oral medicines.

S.N.	Oral Medicine	Dosage	Duration	Ingredients
1.	*Capsule Grab*	1 tablet (20mg) thrice a day after food	1 month	*VranapahariRasa*-100mg, *Triphala guggulu-*300mg *Gandhaka rasayana*-75mg *Arogyavardhini rasa*-25mg *Guduchi*-Q.S. *Manjishta*-Q.S.
2.	*Bilwadi agada gulika*	2 tablets (250mg each tablet) twice a day after food, morning and night	3 months	*Vilvamoola, Surasapushpa, Karanjaphala, Natam,Surahva, Haritaki,Amalaki, Vibhitaki,Sunthi, Maricha,Pippali, Haridra,Daruharidra* each 0.073 g *Bastamutra*-Q.S.
3.	*Gandhaka Rasayana*	One tablet (125mg) thrice a day after food	1 month	*Sudha Gandhaka*-750mg *Twak,Ela,Patra, Nagakesara* each 15mg,*Haritaki, Vibhitaki,Amalaki* each 40mg,*Bhringaraja* 800mg.
4.	*Mahamanjishtadi kashaya*	15ml twice a day after food, morning and night with warm water	3 months	*Manjishta* (Rubia cordifolia), *Mustaka* (Cyperus rotundus), *Karanja beej* (Pongamia pinnata), *Shunthi* (Zingiber officinalis), *Kustha* (Saussurea lappa), *Bharangi* (Clerodendrum serratum), *Haridra* (Curcuma longa), *Daruharidra* (Berberis aristata), *Haritaki* (Terminalia chebula), *Bhibhitaki* (Terminalia bellerica), *Amalaki* (Emblica officinalis) etc.

## 5 Timeline

After the informed consent treatment was initiated from the first day of admission (June 10, 2024).Further treatment was carried out in four schedules. Total duration of the treatment including oral medicine was four months (Table .2).

**Table 2:** Timeline interventions.

S.N.	Date/Month	Intervention
1.	June 10, 2024 to June 19, 2024	Hospital care on admission: • Washing with lemon juice • Panchavalkala kashaya parisheka • Bandha with Jatyadi taila • Oral medicines
2.	June 20, 2024 to July 20, 2024 1st month	Discharged from hospital on June 19, 2024 (treatment continued in home, advised follow up for every 15 days for wound cleaning in hospital) • Washing with lemon juice • Panchavalkala kashaya parisheka • Bandha with Jatyadi taila • Oral medicines
3.	July 21, 2024 to August 21, 2024 2nd month	Home care: • Washing with lemon juice • Panchavalkala kashaya parisheka • Bandha with Jatyadi taila • Oral medicines
4.	August 22, 2024 to September 22, 2024 3rd month	Home care: • Washing with lemon juice • Panchavalkala kashaya parisheka • Bandha with Jatyadi taila • Oral medicines

## 6 Follow up and outcome

After 9 days of treatment in the hospital, the patient was discharged, showing considerable improvement in the ulcer's healing process. A good amount of granulation tissue was evident, and both swelling and pain had decreased significantly. During the first follow-up visit, there was no swelling around the ulcer, and the pain had reduced. By the second follow-up, which was 60 days after starting the treatment, the ulcer had started to heal, with the necrotic tissue being removed during the dressing. At the third follow-up, the ulcer was almost fully healed, leaving only a scar at the affected site. By the fourth follow-up, the area had regained its normal skin color, and there was a complete resolution of edema and pain.

## 7 Discussion

The selection of *Pariseka* and *Prakshalana* is grounded in Ayurvedic principles targeting both detoxification and tissue repair. From the perspective of Agada Tantra Sarpavisha Chikitsa, *Pariseka* helps to dilute and wash away the residual venom toxins, pacifying the aggravated *doshas*. This holistic approach leverages the synergistic effects of detoxification, infection control, and tissue regeneration, ensuring comprehensive management of snakebite-induced chronic ulcers.

Citric acid, derived from lemon (*Citrus limon*), is utilized for its antimicrobial efficacy, particularly in wounds with multi-drug resistant bacterial infections. Fresh lemon, lemon juice, lemon peel, and lemon extract have been reported to have antibacterial and antifungal effects because of its high levels of flavanoids and phenolic contents [9].Citric acid supports healthy granulation tissue formation [10] and the acidic environment created by citric acid lowers pH, inhibiting bacterial growth and thus preventing infections, enhances epithelialization both critical processes in wound healing [11].

*Panchavalkala Kashaya*, is used for the treatment of *Dushta Vrana* [12] (chronic or infected wounds) and has recognized antimicrobial properties that aid in infection control and wound management [13]. Its pharmacological profile includes astringent, and anti-inflammatory properties, confirmed by in vitro and in vivo studies.The tannins and polyphenols present in the formulation are known to exert bacteriostatic effects and aid in protein precipitation, forming a protective layer over the wound bed, which supports tissue regeneration and limits secondary infections. *Bilwadi Agada Gulika* is a well-documented antitoxic formulation in Ayurvedic pharmacopeia. It acts systemically to counteract toxic effects and supports the elimination of residual venom by improving hepatic and renal detoxification processes. A recent pharmacological review highlighted the anti-inflammatory and immunomodulatory potential of key constituents like *Bilva* (*Aegle marmelos*) and *Shyonaka* (*Oroxylum indicum*), both critical in post-envenomation recovery.

Internal medications work at systemic level to neutralize remaining venom effects, enhance immune function, support blood purification and promote healthy tissue generation.

Additionally, *Mahamanjishtadi Kashaya* exerts *Rakta Prasadana* (blood-purifying) and *Vrana Ropana* effects. It is rich in polyphenols, notably from *Rubia cordifolia* (Manjishta), which have demonstrated antioxidant and anti-inflammatory properties. These bioactivities help reduce oxidative stress and support neovascularization and collagen deposition—essential for effective wound healing [14].

It is important to recognize that Ayurvedic management in this case complemented the acute care provided through modern medicine. Anti-snake venom (ASV) remains the cornerstone of acute envenomation management by neutralizing circulating venom. However, ASV does not reverse tissue necrosis or promote wound healing in chronic stages. Moreover, in rural or resource-limited settings, delayed administration or inadequate access to ASV can lead to complications such as chronic ulcers. In this context, Ayurvedic modalities offer valuable adjunctive care by addressing chronic wound pathology, enhancing host tissue repair, and improving overall recovery. Therefore, integrative management combining timely ASV administration with supportive Ayurvedic wound care may offer optimal outcomes in snakebite survivors.

## 8 Conclusion

The ulcer achieved complete healing within four months of treatment following the Ayurvedic protocol. This case study highlights that the careful application of Ayurvedic principles, along with the appropriate selection and combination of drugs, can produce promising outcomes in managing ulcers resulting from viper bites. This approach can also help prevent serious complications, such as the need for amputation.

### 8.1 Patient's perspective

When the patient visited OPD (10-6-2024) with her complaints she described a profound sense of anxiety and fear regarding no significant improvement with the conventional wound care including antibiotics and topical antiseptic applications, the potential for long term disability. By the end of 1st month (29-6-2024) patient noticed reduction in purulent discharge and decrease in pain sensitivity. After the four months (10-10-2024) when the complete closure of the wound achieved, patient reported a full return to normal activity levels and a significant improvement in her overall life. Patient expressed increased optimism and a sense of empowerment due to the visible progress. However patient reported a slight increase in urinary frequency and gradual improvement in bowel regularity which she understood as a sign of body's detoxification process.

### 8.2 Limitation

Swab culture was not taken from the ulcer which would have helped to understand the organism involved and to evaluate the possible antimicrobial effects of the medicines used in the study.

## Funding sources

None.

## Author contributions

**Sandesh Kumar:** Conceptualization, Methodology, Validation, Investigation, Writing – original draft, Writing – review & editing, Supervision. **Sukanya Bhat:** Conceptualization, Methodology, Validation, Resources, Writing – original draft, Writing – review & editing.

## Declaration of generative AI in scientific writing

Have not used any tool/servicer or AI for the preparation of the manuscript.

## Conflict of interest

The authors declare that they have no known competing financial interests or personal relationships that could have appeared to influence the work reported in this paper.
